# Kounis Syndrome Triggered by Intravenous Co-Amoxiclav: A Case of Transient ST Elevation in the Context of Anaphylaxis

**DOI:** 10.7759/cureus.89641

**Published:** 2025-08-08

**Authors:** Yusif Shakanti, Abdulrahman Irhouma, Ahmed Hassan

**Affiliations:** 1 Emergency Medicine, Stockport NHS Foundation Trust, Stockport, GBR; 2 General Medicine, Manchester University NHS Foundation Trust, Manchester, GBR

**Keywords:** allergy and anaphylaxis, co-amoxiclav, intramuscular epinephrine complications, kounis syndrome (ks), st-elevation myocardial infarction (stemi)

## Abstract

Kounis syndrome, also known as allergic myocardial infarction, is a rare but potentially life-threatening condition in which acute coronary events are triggered by an allergic reaction. The pathophysiology involves mast cell degranulation and the release of inflammatory mediators such as histamine, leukotrienes, and platelet-activating factor, leading to coronary vasospasm, myocardial ischemia, or infarction. We present the case of a female patient in her 80s with no prior history of coronary artery disease who developed anaphylaxis shortly after intravenous administration of co-amoxiclav in the emergency department. She had no documented allergy to penicillin or prior hypersensitivity reactions. Within minutes, she experienced an acute onset of dyspnoea, hypotension (systolic BP dropped to 70 mmHg), and widespread urticaria. The patient was treated promptly with intramuscular epinephrine (0.5 mg), leading to hemodynamic stabilization and resolution of ECG changes. She was admitted for observation and recovered without further cardiac complications. Simultaneously, her ECG done within 15 minutes of the onset of the allergic reaction showed new ST-segment elevation in the inferior leads and anterior leads. She went on to have serial ECGs performed to monitor disease progression, and the ST elevations showed partial resolution within 30 minutes following treatment, with complete resolution 12 hours later. High-sensitivity cardiac troponin I was initially 10 ng/L (0-54 ng/L) and remained essentially unchanged at 23 ng/L after 12 hours, suggesting a non-dynamic pattern on serial monitoring. Echocardiography showed normal left ventricular function without regional wall motion abnormalities. Coronary angiography was not performed, as the clinical presentation, rapid normalization of ECG changes, normal echocardiographic findings, and non-dynamic cardiac biomarkers strongly supported a vasospastic rather than obstructive coronary pathology. This case illustrates the diagnostic challenge of differentiating Kounis syndrome from typical acute coronary syndromes, particularly in older adults. Recognizing the allergic trigger and observing the transient nature of ECG changes can help avoid unnecessary invasive procedures. Management should focus on treating the allergic reaction, which may be sufficient to reverse myocardial involvement. Kounis syndrome should be considered in patients presenting with ECG changes following an allergic reaction. Treatment should prioritize the management of the hypersensitivity response, which may in fact reverse cardiac involvement without the need for invasive cardiac procedures.

## Introduction

Anaphylaxis is a severe, life-threatening systemic hypersensitivity reaction that can occur rapidly following exposure to allergens. Kounis syndrome, also referred to as allergic acute coronary syndrome, is a rare but increasingly recognized condition in which an allergic reaction triggers myocardial ischemia. Kounis syndrome occurs in approximately 1.1% of patients hospitalised with allergic or anaphylactic reactions in large cohort studies in the United States [1]. The pathophysiology involves mast cell degranulation and the release of inflammatory mediators such as histamine, leukotrienes, and platelet-activating factor, which induce coronary artery vasospasm leading to myocardial injury [2-4].

Kounis syndrome typically presents with chest pain alongside signs and symptoms of a systemic allergic reaction, such as urticaria, bronchospasm, wheezing, or airway compromise; ST-segment elevation or depression is often seen on ECG, and cardiac biomarkers may be elevated in some cases [5]. It is classified into three types. Type I, observed in patients with no underlying coronary artery disease, results from coronary vasospasm caused by the allergic cascade. Type II occurs in individuals with pre-existing coronary atherosclerosis, where allergic inflammation can provoke plaque rupture or erosion. Type III involves stent thrombosis due to hypersensitivity reactions [2]. Kounis syndrome is more often described in younger individuals [6]. 

Co-amoxiclav, a widely prescribed combination of amoxicillin and clavulanic acid, is a known cause of drug-induced hypersensitivity reactions such as anaphylaxis [7]. Transient ST elevation in the setting of anaphylaxis strongly supports the diagnosis of Kounis syndrome [8,9].

In this report, we present the case of a patient who was in her 80s, an atypical age group for Kounis syndrome, highlighting the importance of recognising that age alone does not preclude the diagnosis, and elderly patients may still present with Type I Kounis syndrome. In this case, the diagnosis of Type I Kounis syndrome was supported by the absence of chest pain, rapid ECG normalisation, minimal and non-dynamic troponin rise, and no evidence of regional wall motion abnormalities on echocardiography. Although the patient was elderly, these findings pointed toward transient coronary vasospasm in the absence of plaque rupture. The patient had previously tolerated flucloxacillin; however, she developed anaphylaxis within minutes of receiving intravenous co-amoxiclav, suggesting clavulanic acid as the likely allergenic trigger. During the anaphylactic episode, she developed ST-segment elevation on her electrocardiogram (ECG), which is a significant finding pointing to Kounis syndrome.

## Case presentation

A female patient in her 80s presented to the emergency department with worsening left lower limb cellulitis despite completing a course of oral antibiotics. She was fully alert and oriented at the time of arrival. Her past medical history included hypertension, chronic kidney disease stage 3, pernicious anemia, atrial fibrillation, primary hyperparathyroidism, and a previous ischemic stroke. Her regular medications included alendronate 70 mg once weekly, atorvastatin 20 mg once daily, hypromellose eye drops, edoxaban 30 mg once daily, metoprolol 200 mg once daily, and senna as required.

Following clinical assessment, the decision was made to escalate antibiotic therapy to intravenous co-amoxiclav. A single dose of 1.2 g was administered intravenously. Within two minutes of administration, the patient developed acute respiratory distress with stridor and sudden unresponsiveness. The emergency buzzer was activated by nursing staff, and the patient was immediately transferred to the resuscitation area for assessment and management.

On arrival in resus, she was administered 15 L of oxygen via a non-rebreather mask. Initial observations revealed oxygen saturation of 76%, respiratory rate of 24 breaths per minute, heart rate of 60 breaths per minute, blood pressure of 67/46 mmHg, and a temperature of 35.3°C. Clinical examination confirmed severe respiratory distress, stridor, a silent chest on auscultation, and visibly engorged neck veins. Airway manoeuvres were attempted, with partial improvement. Given the rapid deterioration and temporal relationship to co-amoxiclav administration, intramuscular epinephrine 0.5mg was administered into the anterolateral thigh for suspected anaphylaxis.

**Figure 1:**
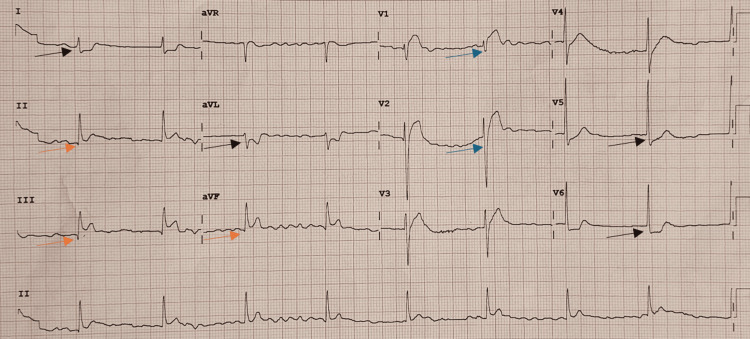
12-lead ECG recorded within 15 minutes of symptom onset during an anaphylactic reaction showing ST-segment elevation. Marked ST-segment elevations are visible in the inferior leads (orange arrow, II, III, aVF) and anterior leads (blue arrow, V1–V2) with ST-segment depression in the lateral leads (black arrow, I, aVL, V5-V6).

Within 15 minutes, an ECG performed as part of her workup showed ST-segment elevation in both inferior (II, III, aVF) and anterior (V1-V2) leads, along with ST depression in the lateral leads (V5, V6, I, and aVL), raising concern for myocardial infarction (Figure 1).

As treatment continued, the patient’s condition began to improve. She became responsive to pain, and her breathing stabilized. A focused bedside echocardiogram showed good biventricular function with no regional wall motion abnormalities.

**Figure 2:**
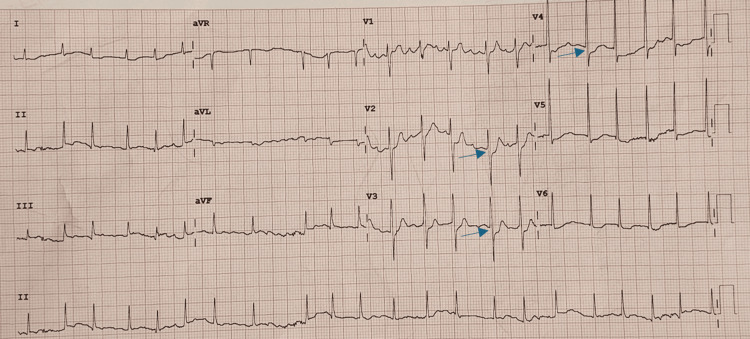
ECG 30 minutes post onset of anaphylaxis demonstrating partial resolution of ST changes 12-lead ECG recorded 30 minutes after the onset of anaphylaxis. Compared to the initial ECG, there is resolution of ST elevation in the inferior leads and some new ST depression in leads V2-V4. These dynamic changes further support a diagnosis of Type I Kounis syndrome, characterised by transient myocardial ischemia due to coronary vasospasm rather than fixed coronary obstruction. Blue arrows highlight areas of ST depression changes.

Approximately 10 minutes after the initial ECG (20 minutes since the onset of symptoms), she developed widespread urticaria and wheezing. Her consciousness level continued to improve, and she was able to speak in short sentences. A repeat ECG performed 15 minutes after the initial ECG showed partial resolution of the ST-segment changes previously observed, with some new ST-segment depression in leads V2-V4 (Figure 2). 

**Figure 3:**
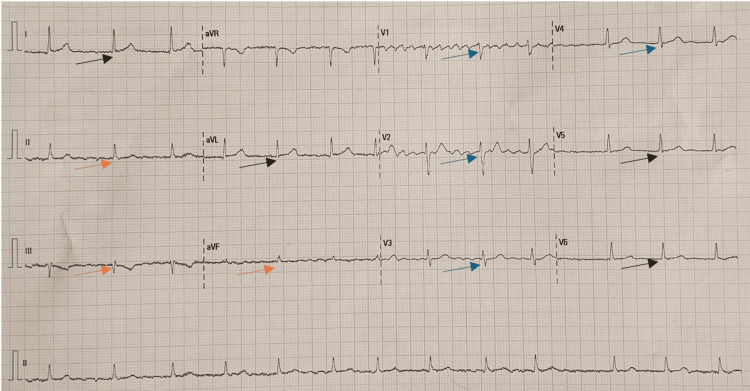
ECG 12 hours after the onset of the anaphylactic reaction showing complete resolution of all ST-segment changes Arrows are pointing to ST-segments showing resolution of ischemia, black arrows show lateral leads (I, aVL,V5,V6), orange arrows show inferior leads (I,II,aVF) and blue arrows show anterior leads (V1-V4).

Twelve hours later, a repeat ECG was performed, which showed complete resolution of all previous ST-segment changes (Figure 3).

On presentation to the Emergency Department, blood tests were performed. These included a full blood count, urea and electrolytes, and C-reactive protein. Following the administration of co-amoxiclav, additional requests for mast cell tryptase and troponin were added to the existing sample to establish baseline levels. Repeat measurements of tryptase and troponin were subsequently taken at defined intervals (Table 1).

**Table 1:** Serial blood test results in relation to timing of anaphylactic reaction, including inflammatory markers, cardiac biomarkers, renal function, and eosinophil response. This table outlines key laboratory investigations taken before and after the patient developed an anaphylactic reaction. The patient was initially admitted for lower limb cellulitis, for which baseline inflammatory markers and renal function were assessed. The C-reactive protein (CRP) was elevated (61.6 mg/L), consistent with infection. Following the onset of anaphylaxis, mast cell tryptase levels were measured and showed a rise from baseline (3.1 µg/L) to a peak of 13.4 µg/L at three hours, then declined at 12 hours, a pattern strongly supportive of mast cell activation and anaphylaxis. A mild transient rise in high-sensitivity troponin-I (from 10 ng/L to 23 ng/L) was observed over 12 hours, indicating myocardial stress. Additionally, eosinophil count rose from 0.2 to 0.6 × 10⁹/L, supporting an allergic etiology. These findings support a diagnosis of Type I Kounis syndrome, where allergic-mediated coronary vasospasm resulted in transient myocardial injury. CRP: C-reactive protein, eGFR: estimated glomerular filtration rate

Test	Timing relative to anaphylactic reaction	Result	Reference Range
Mast cell tryptase	2 hours before	3.1 µg/L	0–12.9 µg/L
	3 hours after	13.4 µg/L	
	12 hours after	4.6 µg/L	
High sensitivity troponin-I	2 hours before	10 ng/L	0–54 ng/L
	12 hours after	23 ng/L	
Eosinophils	2 hours before	0.2 × 10⁹/L	0–0.6 × 10⁹/L
	3 hours after	0.6 × 10⁹/L	
White cell count	2 hours before	6.6 × 10⁹/L	3.7–11.0 × 10⁹/L
CRP	2 hours before	61.6 mg/L	<10 mg/L
eGFR	2 hours before	48 mL/min/1.73 m²	>60 mL/min/1.73 m²
Haemoglobin	2 hours before	101 g/L	115–165 g/L

In the end, the patient was treated promptly with a single intramuscular dose of 0.5 mg epinephrine. She also received high-flow oxygen via a 15L non-rebreather mask and intravenous fluids to address hypotension. Coronary angiography was not performed, as the clinical presentation, rapid normalization of ECG changes, normal echocardiographic findings, and non-dynamic cardiac biomarkers strongly supported a vasospastic rather than obstructive coronary pathology. No further troponin levels were taken beyond 12 hours, as the modest peak of 23 ng/L at the 12-hour mark, in the context of clinical improvement and resolving ECG changes, was not consistent with plaque rupture or evolving myocardial infarction.

The patient was evaluated by Cardiology during her inpatient stay in the hospital, and it was agreed that this is not in keeping with a plaque rupture acute coronary syndrome. Transthoracic echocardiography was performed three days after admission. It demonstrated borderline low left ventricular systolic function with an estimated ejection fraction of approximately 50%, moderate aortic stenosis, and no evidence of regional wall motion abnormalities. There was no prior echocardiogram available for comparison.

The patient was treated with clindamycin, which is considered safe in individuals with a suspected allergy to co-amoxiclav, for the remainder of her hospital stay to manage cellulitis. She remained an inpatient for five days, and her recovery was uneventful. Penicillin and co-amoxiclav were documented as allergies on her drug chart, and this was communicated to her general practitioner in the discharge summary. Upon discharge, she was also referred to a tertiary allergy clinic for further evaluation and investigation. 

## Discussion

Kounis syndrome occurs when the response to the chemical mediators of anaphylaxis results in transient coronary vasospasm, myocardial infarction, or coronary stent thrombosis [2-4,6]. This case presented several diagnostic challenges; however, a number of key findings helped establish the diagnosis of Kounis syndrome. 

First, the clinical context was highly suggestive of anaphylaxis. The patient developed airway compromise requiring airway manoeuvres, along with severe hypoxia and wheeze, all occurring within minutes of intravenous co-amoxiclav administration. It is well recognised that anaphylaxis progresses more rapidly and with greater severity following intravenous exposure compared to other routes, often within minutes, increasing the risk of fatal outcomes [8].

Importantly, ST-segment elevation on the ECG was observed prior to any meaningful haemodynamic recovery or significant systemic distribution of exogenously administered epinephrine. This temporal relationship strongly suggests that the ECG changes occurred at the peak of the allergic reaction and were not caused by epinephrine administration and, therefore, were not iatrogenic in nature. While other case reports have implicated epinephrine as a potential cause of myocardial ischaemia [9], the clinical context in this case supports an alternative mechanism, namely, coronary vasospasm due to anaphylaxis consistent with Kounis syndrome.

At this stage, the administration of epinephrine requires careful clinical judgment, as it may exacerbate myocardial ischaemia and precipitate life-threatening arrhythmias. Nevertheless, it remains the cornerstone of anaphylaxis management and should not be withheld when clinically indicated, as in this case. 

To further evaluate the cause, a repeat ECG and bedside transthoracic echocardiogram were performed. The ECG changes resolved rapidly without the need for percutaneous coronary intervention, which is more consistent with Type I Kounis syndrome, transient coronary vasospasm, likely mediated by histamine or leukotrienes, rather than acute myocardial infarction or prolonged vasospasm from epinephrine toxicity. The echocardiogram demonstrated good biventricular function with no regional wall motion abnormalities, further supporting the diagnosis.

The patient had received 0.5 mg of intramuscular epinephrine, the standard and recommended first-line dose for anaphylaxis. At this dose and route, epinephrine is rarely associated with myocardial ischaemia, except in cases of overdose or intravenous administration, which may cause arrhythmias or intense vasoconstriction [10].

At the time, the clinical team suspected allergic myocardial ischaemia consistent with Type I Kounis syndrome. This diagnosis was further supported the following day by laboratory results. Serum tryptase levels rose from 3.1 µg/L at baseline to 13.4 µg/L three hours post exposure, confirming mast cell activation and supporting a diagnosis of anaphylaxis [11]. In addition, high-sensitivity troponin I levels showed only a mild, non-dynamic elevation, which did not follow a pattern typical of myocardial infarction, further supporting a diagnosis of Type I Kounis syndrome [12]. The prompt initiation of treatment likely mitigated prolonged coronary vasospasm and helped minimise myocardial tissue injury. Furthermore, serial ECGs demonstrated resolving changes, and echocardiography showed no regional wall motion abnormalities, all of which were consistent with transient coronary vasospasm from Kounis syndrome type I rather than obstructive coronary artery disease plaque rupture.

In the context of an allergic reaction, both Type I and Type II Kounis syndrome should be considered as potential causes of ST-segment changes on ECG, with Type II being particularly relevant in elderly patients due to their higher likelihood of underlying atherosclerotic disease. If ECG changes persist, early consultation with the primary percutaneous coronary intervention (PPCI) service is warranted. However, when ECG abnormalities are transient, clinicians should consider coronary vasospasm due to Kounis syndrome type I as the likely underlying mechanism. When there is a strong clinical suspicion of anaphylaxis, it must be treated promptly, in parallel with cardiac evaluation.

Given the underlying mechanism of Type I Kounis syndrome, transient coronary vasospasm in the absence of plaque rupture, coronary angiography was not pursued, as it was unlikely to offer additional diagnostic or therapeutic benefit. However, in cases of suspected Type II or Type III Kounis syndrome, particularly when there is a dynamic rise in troponin and persistent or evolving ECG changes, coronary angiography would be warranted to assess for obstructive coronary artery disease or plaque rupture [13].

We recommend that suspected drug allergies, particularly in cases of Kounis syndrome, be clearly documented in the patient’s medical records to prevent future exposure. Additionally, we propose that future guidelines emphasise the value of multidisciplinary input, integrating both allergy and cardiology specialists at appropriate stages of assessment and management.

## Conclusions

This case highlights the potential for commonly prescribed antibiotics such as co-amoxiclav to trigger severe allergic reactions, including anaphylaxis, which may lead to Kounis syndrome. Early recognition and appropriate management of anaphylaxis are critical to preventing serious complications or death. Clinicians should maintain a high index of suspicion for drug-induced anaphylaxis and its cardiovascular manifestations, particularly Kounis syndrome, as misdiagnosis can significantly alter both management and patient outcomes.

This case underscores the importance of clinician education and awareness in recognising ECG changes that may be allergic in origin. Serial ECGs and cardiac biomarkers are essential in distinguishing transient coronary vasospasm, characteristic of Type I Kounis syndrome, from evolving myocardial infarction seen in Type II and Type III variants. When available, bedside echocardiography performed by a trained practitioner can offer valuable diagnostic insight by identifying regional wall motion abnormalities and supporting a non-obstructive cause of myocardial injury. Importantly, patients with Type I Kounis syndrome often do not require coronary intervention, as the underlying mechanism involves transient vasospasm rather than fixed atherosclerotic obstruction.
